# Enhanced Th17 responses in the appendix of children with complex compared to simple appendicitis are associated with microbial dysbiosis

**DOI:** 10.3389/fimmu.2023.1258363

**Published:** 2024-01-04

**Authors:** Sarah-May M. L. The, Renée R. C. E. Schreurs, Agata Drewniak, Roel Bakx, Tim G. J. de Meij, Andries E. Budding, Linda Poort, Huib A. Cense, Hugo A. Heij, L. W. Ernest van Heurn, Ramon R. Gorter, Madeleine J. Bunders

**Affiliations:** ^1^ Department of Paediatric Surgery, Emma Children’s Hospital, Amsterdam University Medical Center (UMC), University of Amsterdam & Vrije Universiteit Amsterdam, Amsterdam, Netherlands; ^2^ Amsterdam Reproduction and Development Research Institute, Amsterdam, Netherlands; ^3^ Department of Experimental Immunology, Amsterdam Infection & Immunity Institute, Amsterdam UMC, University of Amsterdam, Amsterdam, Netherlands; ^4^ Department of Paediatrics, Emma Children’s Hospital, Amsterdam UMC, University of Amsterdam & Vrije Universiteit Amsterdam, Amsterdam, Netherlands; ^5^ Amsterdam Gastroenterology and Metabolism Research Institute, Amsterdam, Netherlands; ^6^ Department of Paediatric Gastroenterology, Emma Children’s Hospital, Amsterdam UMC, University of Amsterdam, Amsterdam, Netherlands; ^7^ inBiome, Amsterdam, Netherlands; ^8^ Department of Surgery, Red Cross Hospital, Beverwijk, Netherlands; ^9^ Leibniz Institute of Virology, Hamburg, Germany; ^10^ Third Department of Medicine, University Medical Center Hamburg-Eppendorf, Hamburg, Germany

**Keywords:** appendicitis, children, T cells, Th17, microbiota

## Abstract

**Introduction:**

Appendicitis is one of the most common causes of acute abdominal surgery in children. The clinical course of appendicitis ranges from simple to complex appendicitis. The mechanisms underlying the heterogeneity of appendicitis in children remain largely unclear. Dysregulated T cell responses play an important role in several inflammatory diseases of the intestine, but the extend of T cell dysregulation in appendicitis in children is less well known.

**Methods:**

To characterize appendiceal T cells in simple and complex appendicitis we performed in-depth immunophenotyping of appendiceal-derived T cells by flow cytometry and correlated this to appendiceal-derived microbiota analyses of the same patient.

**Results:**

Appendix samples of twenty children with appendicitis (n = 8 simple, n = 12 complex) were collected. T cells in complex appendicitis displayed an increased differentiated phenotype compared to simple appendicitis, including a loss of both CD27 and CD28 by CD4^+^ T cells and to a lesser extent by CD8^+^ T cells. Frequencies of phenotypic tissue-resident memory CD69^+^CD4^+^ T cells and CD69^+^CD8^+^ T cells were decreased in children with complex compared to simple appendicitis, indicating disruption of local tissue-resident immune responses. In line with the increased differentiated phenotype, cytokine production of in particular IL-17A by CD4^+^ T cells was increased in children with complex compared to simple appendicitis. Furthermore, frequencies of IL-17A^+^ CD4^+^ T cells correlated with a dysregulation of the appendiceal microbiota in children with complex appendicitis.

**Conclusion:**

In conclusion, disruption of local T cell responses, and enhanced pro-inflammatory Th17 responses correlating to changes in the appendiceal microbiota were observed in children with complex compared to simple appendicitis. Further studies are needed to decipher the role of a dysregulated network of microbiota and Th17 cells in the development of complex appendicitis in children.

## Introduction

Appendicitis is the most common inflammatory cause of emergency abdominal surgery in children (1). Historically, appendicitis was assumed to be an irreversible disease caused by intraluminal obstruction with progression into perforation over time. Nowadays, the pathogenesis of appendicitis is considered to be more heterogeneous, with changes in microbiota, tissue perfusion and pro-inflammatory immune responses (2, 3). The exact underlying mechanisms however remain incompletely understood. Emerging data suggests that some children develop a milder appendicitis (phlegmonous i.e., simple appendicitis) which can be resolved by conservative treatment with antibiotics (3–5), whereas other children rapidly progress to complex appendicitis (gangrenous or perforated appendicitis) which often requires immediate surgery (3, 6). To develop tailored strategies for children with simple and complex appendicitis, a better understanding of the underlying inflammatory processes in the appendix is needed.

The appendix itself extends from the caecum and is enriched in immune cells resulting in gut-associated lymphoid tissue similar to Peyer’s patches (7, 8). The appendix is suggested to function as a safe house for commensal bacteria, which together with the enrichment of immune cells allows for a critical role of the appendix in shaping intestinal immune responses under homeostatic and inflammatory conditions (7, 9–11). Previous studies suggest that CD4^+^ T cells and CD8^+^ T cells are affected in the appendix during inflammation and may differ in frequencies between children with simple and complex appendicitis (12, 13).

Dysregulated T cell responses have been shown to play a critical role in the development of several other inflammatory diseases: A decrease in regulatory T cells (Tregs) and increase of Th1/Th17 cells has been shown to contribute to inflammation in inflammatory bowel disease (IBD) (14–16). Furthermore, tissue-resident memory T cells (Trm) play a critical role in protection against pathogens, but when dysregulated mediate inflammation (17–19). In particular, enriched populations of Th17 Trm cells have been identified in patients with active Crohn’s disease (20, 21). Moreover, in the context of pediatric diseases we have demonstrated that the expansion of tissue necrosis factor (TNF) producing CD4^+^ T cells in preterm children can contribute to necrotizing enterocolitis (22). Taken together, dysregulated T cell responses importantly contribute to other inflammatory diseases in children, but the potential role of appendiceal T cells remains largely unclear.

Here, we investigated appendiceal tissues derived from children with simple and complex appendicitis who underwent an appendectomy. These analyses showed a disruption of local T cell responses, and increased frequencies of effector IL-17A-producing CD4^+^ T cells in the appendix of children with complex compared to simple appendicitis. Paired microbiota analyses showed that Proteobacteria were positively correlated with IL-17A production and *Ruminococcus* sp. were negatively correlated, indicating a dysregulated microbiota-T cell network in children with complex appendicitis.

## Materials and methods

Between November 2015 and November 2016, samples were collected in the context of the study performed at the Amsterdam University Medical Center (UMC), Amsterdam and at the Red Cross Hospital, Beverwijk, The Netherlands. The medical ethics committee of the Amsterdam UMC approved the study, with local permission granted by the medical board of the Red Cross Hospital, Beverwijk. Children (0-17 years old) with suspected appendicitis that underwent an appendectomy were eligible for inclusion. Written informed consent was obtained from parents/legal guardians of all children and from children of twelve years and older. Children were excluded if a diagnosis other than appendicitis (such as, but not limited to, inflammatory bowel disease or malignancy of the appendix) was suspected or confirmed. Use of prophylactic antibiotics prior to surgery was not an exclusion criterion as it is standard surgical care for acute appendicitis.

### Sample collection

All children underwent open or laparoscopic appendectomy and received prophylactic antibiotics according to local protocol (~ 30 minutes before incision): 1) metronidazole (<12 years 7.5 mg/kg/dose iv, maximum of 500 mg/dose; ≥12 years 500 mg/dose iv) in combination with cephalosporin (cefazolin 30-50 mg/kg/dose, maximum of 2 g/dose or cefuroxime 50 mg/kg/dose, maximum of 1.5 g/dose), or 2) amoxicillin-clavulanic acid (<40 kg 100/10 mg/kg/day in 3-4 doses; >40 kg 1000/100 to 2000/200 mg/dose iv) in combination with gentamicin (7 mg/kg/day). After appendectomy, the larger tissue part of the appendix was used for routine histopathologic evaluation. In addition, one cm of the appendix was used for the study. Half of the tissue collected for the study was stored at 4°C and immune cells were isolated within 36 hours. The remaining half of the tissue was stored immediately after surgical removal at -20°C and used for analysis of appendiceal microbiota.

### Clinical and histology classification

Samples of the appendix of twenty children with confirmed appendicitis were obtained for immunological analysis. To classify the cases in simple and complex appendicitis, two authors, both blinded for immunological and microbiota results, performed the classification of the patients using predefined intraoperative and histopathological data. Simple (i.e., uncomplicated) appendicitis was defined as phlegmonous appendicitis without signs of complexity, and complex appendicitis as gangrenous or perforated appendicitis, with signs of excessive ulceration or necrosis, with or without abscess formation. In case of disagreement, a third author was consulted to reach a final decision.

### Cell isolation

Mononuclear cells were isolated from tissue as previously described (22, 23). Briefly, the tissue size was measured and split into a mucosal and muscular layer with scissors. The epithelial cells were then separated from the mucosa by incubation with a mixture of Iscove’s Dulbecco’s Medium (IMDM; Lonza; Cat# BE12-722F), 1% Fetal Bovine Serum (FBS; Biological Industries; Cat# 04-007), 5mM ethylenediaminetetraacetic acid (EDTA; Sigma-Aldrich; Cat#03690; CAS: 60-00-4), 2mM 1,4-dithiothreitol (DTT; Sigma-Aldrich; Cat# D8255; CAS: 6892-68-8) and 200 U/ml DNAse type I (Roche; Cat# 10104159001). Isolation of cells from the lamina propria and muscular layer was performed through enzymatic digestion. In short, each layer was minced and incubated with a 10 ml Collagenase D mixture: IMDM (Lonza), 1 mg/ml (0.15 U/g) Collagenase D (Roche; Cat# 11088866001; EC: 3.4.24.3), FBS (Biological Industries) and 1000 U/ml DNAse type 1 (Roche). Cells harvested from the three layers were filtered through a 70 um cell strainer (Falcon, Corning; Cat# 352350) to obtain a single cell suspension.

Then, to retrieve a mononuclear cell fraction from the separate single cell suspensions, density gradient separation was performed. All cell suspensions were dissolved in 10 ml Hank’s Balanced Salt Solution (HBSS) (Lonza; Cat# BE10-543F) and layered on a Lymphoprep (Axis-Shield; Cat# 1114547) gradient for the epithelial cells and a custom Percoll gradient (Sigma-Aldrich; Cat# GE17-0891-01) for the cells of the lamina propria and muscular layer. Samples were then centrifuged and interphases containing mononuclear cells were collected. The counting of viable cells was performed with 10 ul trypan blue staining (Sigma-Aldrich; Cat# T8154; CAS: 72-57-1) in a Bürker chamber.

### T cell characterization using flow cytometry

Mononuclear cells were incubated with their respective antibody mixes for thirty minutes in the dark at 4°C, and washed and fixated using 1X stabilizing fixative (Thermo Fisher Scientific; Cat# 00-5123-43) as previously described (22, 23). Staining was performed using antibodies directed against (all anti-human) CD45 (FITC; HI30; Thermo Fisher Scientific, Cat# 11-0459-42; RRID: AB_10852703), CD45 (BV711; HI30; Biolegend; Cat# 304049; RRID: AB_2563465), CD3 (V500; UCHT1; BD Biosciences; Cat# 561416; RRID: AB_10612021), CD4 (BUV737; SK3; BD Biosciences; Cat# 564305; RRID: AB_2713927), CD8a (BV785; RPA-T8; Biolegend; Cat# 301045; RRID: AB_11219195), CD45RA (BV650; HI100; BD Biosciences; Cat# 563963; RRID: AB_2738514), CCR7 (BUV395; 150503; BD Biosciences; Cat# 563977; RRID: AB_2738519), CD27 (APC-eFluor780; O323; Thermo Fisher Scientific, Cat# 47-0279-42; RRID: AB_1272040) CD28 (PE; 28.2; Thermo Fisher Scientific, Cat# 12-0289-42; RRID: AB_2016668), CD103 (PerCP-eFluor710; BerACT8; Thermo Fisher Scientific; Cat# 46-1037-41; RRID: AB_11039409), CD25 (APC; BC96; Thermo Fisher Scientific; Cat# 17-0259-41; RRID: AB_1582220), CD69 (BV421; FN50; BD Biosciences; Cat# 562883; RRID: AB_2737863) and CD127 (PE-Cy7;eBioRDR5; Thermo Fisher Scientific; Cat# 25-1278-41; RRID: AB_1659675). Live/Dead viability dye (Fixable Red; Invitrogen; Cat# L23102) was used to assess cell viability. Flow cytometry was performed within 24 hours work-up of individual samples using a LSR Fortessa Flow Cytometer (BD Biosciences). Ultracomp eBeads (Thermo Fisher Scientific; Cat# 01-2222-42) were used to correct for spectral overlap. The flowcytometry data was analyzed with Flowjo software (Treestar; Version V10.5.0; RRID: SCR_008520).

### Cytokine production by T cells

To determine cytokine production of T cells, the cells from the cell suspensions were resuspended in IMDM (with 10% FBS, 50 ug/ml Gentamicin (Gibco; Cat# 15710-049) and 60 uM 2-mercaptoethanol (Sigma-Aldrich; Cat# 516732; CAS: 60-24-2)). Half of the cells were stimulated with 10 ng/ml phorbol 12-myristate 13-acetate (PMA;Sigma-Aldrich; Cat# P8139; CAS: 16561-29-8) and 1 ug/ml ionomycin (io;Sigma-Aldrich; Cat# I0634; CAS: 56092-82-1) (P/I-stimulation), and the other half left untreated as control (unstimulated). After one hour (of stimulation), 7 ug/ml Brefeldin A (Invitrogen; Cat# B7651; CAS: 20350-15-6) was added followed by an additional 12-14 hours of stimulation at 37°C and 5% CO2. Surface staining of the samples was similarly performed as described in the previous paragraph and antibodies directed against Live-dead (Fixable Red;Invitrogen; Cat# L23102), CD45 (BV711; HI30; Biolegend; Cat# 304049; RRID: AB_2563465), CD45 (BV421; HI30; BD Biosciences; Cat# 563879, RRID : AB_2744402), CD3 (V500; UCHT1; BD Biosciences; Cat# 561416; RRID: AB_10612021), CD4 (BUV737; SK3; BD Biosciences; Cat# 564305; RRID: AB_2713927), CD8a (BV785; RPA-T8; Biolegend; Cat# 301045; RRID: AB_11219195) were used, followed by intracellular staining. To this end, cells were washed and fixated with 1X Fixation/Permeabilization reagent (Thermo Fisher Scientific; Cat# 00-5123-43) for 15 minutes, then resuspended in Permeabilization Buffer (Thermo Fisher Scientific; Cat# 00-8333-56) and incubated for thirty minutes with antibodies in the dark at 4°C. Intracellular antibodies (all anti-human) directed against TNF (BUV395; Mab11; BD Biosciences; Cat# 563996; RRID: AB_2738533), IFN-γ (FITC; 4S.B3; Thermo Fisher Scientific; Cat# 11-7319-82; RRID: AB_465415), IL-17A (APC-eFluor780; eBio64DEC17; Thermo Fisher Scientific; Cat# 47-7179-42; RRID: AB_11043559) and IL-2 (BV421; 5344.111; BD Biosciences; Cat# 562914; RRID: AB_2737888) were used. Flow cytometry analyses were performed as described above. T cell viability after 12-14 hours incubation in unstimulated condition (+ Brefeldin A only) was similar to conditions with PMA/Ionomycin stimulation (+ Brefeldin A) (data not shown). For the analyses of CD4^+^ T cells after P/I stimulation, cells were identified as CD3^+^CD8^-^CD4^+/-^ as P/I-stimulation downregulates the expression of CD4. CD8^+^ T cells were identified as CD3^+^CD8^+^CD4^-^ (23).

### Microbiota analysis

The appendices used for T cell analyses above were also analyzed for their local microbial composition of the appendix (24). Combining the data of T cell immunophenotyping and microbiome analyses in the same patient allowed for unique paired analyses of T cells and microbiota. In brief, microbiota analysis was performed using IS-pro, based on 16S-23S rDNA interspace region analysis, performed by InBiome, Amsterdam, The Netherlands (25). After isolation and amplification assays (GeneAmp PCR system 9700), bacteria were classified into the three main phyla: Firmicutes, Actinobacteria, Fusobacteria and Verrucomicrobia (FAFV); Bacteroidetes; and Proteobacteria.

The obtained IS-profiles led to the identification of species (using their length of 16S-23S rDNA regions reflected by taxonomic units or OTU’s) and their relative abundance (by relative fluorescence units; RFU’s). For the purpose of this study overall abundance of the three main phyla and several abundant species, as identified previously, were compared (using their RFU’s) to the production of interleukins by appendiceal CD4^+^ T cells of patients (24). Evaluation of concomitant enteric infections is not included in the standard diagnostic work-up in children suspected of appendicitis, nor is it routinely investigated perioperative in children with confirmed diagnosis of appendicitis.

### Statistical analyses

Graphpad Prism was used to perform statistical analyses and visualization (Graphpad Software; Version 8; RRID : SCR_002798). Fisher’s exact test was used for dichotomous variables and Student’s T test, Mann-Whitney U test and ANOVA test were used where appropriate for continuous variables. For the correlation of microbiota analysis with interleukin production, Spearmann correlations were performed. Statistical significance for all analyses was determined as α < 0.05.

## Results

Appendiceal tissue samples were obtained from twenty children with a confirmed diagnosis of appendicitis: Simple appendicitis, n = 8 (40%) and complex appendicitis n = 12 (60%). Of 18 children enough viable cells were detected in final analyses of T cells. Baseline characteristics are listed in **Table 1**. C-reactive protein (CRP) and blood leukocyte count were significantly higher in children with complex compared to simple appendicitis.

**Table 1:** Baseline characteristics of children with simple and complex appendicitis.

	All	Simple	Complex	P-value
**Number of patients**	20	8	12	
**Age, years**	11 [1-16]	10 [7-16]	12 [1-16]	0.58
**Biological female sex**	11 (55)	5 (63)	6 (50)	0.67
**Days of abdominal pain**	1 [1-8]	1 [1-4]	2 [1-8]	0.40
**Temperature, °C**	37.6 [36.0-40.0]	36.8 [36.3-37.9]	37.9 [36.0-40.0]	0.09
**C-reactive protein, mg/L**	25 [7-139]	18 [11-42]	54 [7-139]	0.03
**Leukocyte count, x10^9^/L**	16.0 [6.6-25.5]	11.0 [6.6-21.0]	17.2 [11.8-25.5]	0.02

Results are presented as median with [range min-max], except for sex which is presented as number of patients and (%). All Mann-Whitney U comparisons, except for sex which is evaluated with Fisher’s Exact.

### Mucosal cytopenia in the appendix of children with complex appendicitis

**Figure 1:**
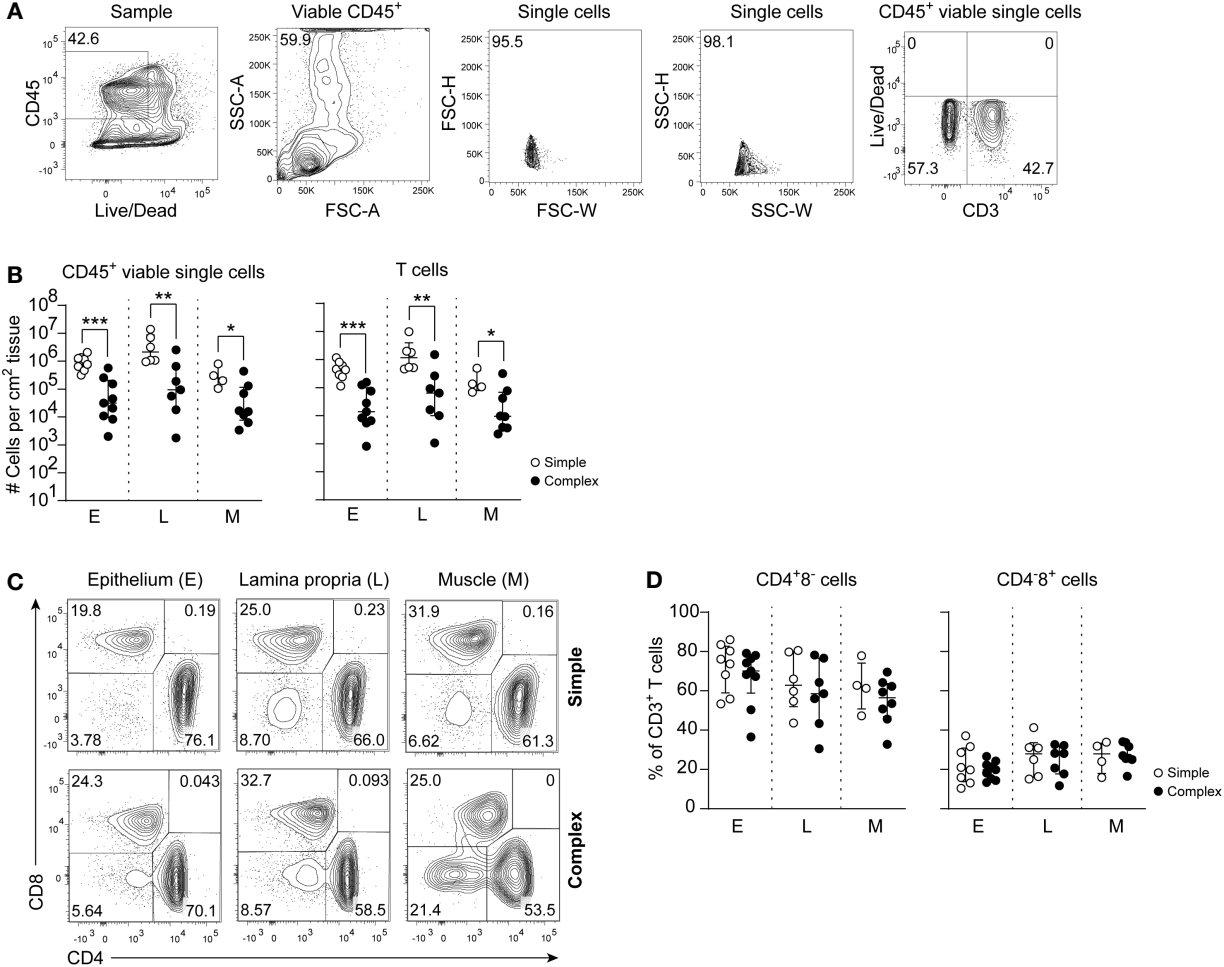
Disease severity is associated with a decrease of CD45^+^ viable cells and specifically T cells in complex appendicitis in children. **(A)** Gating strategy to determine intestinal CD45^+^ viable single cells and T cells (CD3^+^ cells). **(B)** Absolute cells count per cm^2^ of CD45^+^ viable single cells and T cells in the epithelium (E), lamina propria (L) and muscular (M) layers. **(C)** Identification of CD4 and CD8 expressing CD3^+^ T cells. **(D)** Frequencies (%) of CD4^+^ T cells and CD8^+^ T cells in all tissue layers. **(B, D)** represent appendiceal epithelium (simple appendicitis n = 8 and complex appendicitis n = 9), lamina propria (simple appendicitis n = 7 and complex appendicitis n = 7) and muscular layer (simple appendicitis n = 4 and complex appendicitis n = 8). Representative flow cytometric plots are shown. Errors bars represent median percentage with ± interquartile range (IQR). All Mann-Whitney U comparisons. *P <.05, **P <.01, ***P <.001.

Macroscopic analyses of the twenty tissue samples identified distinct features of necrosis, hemorrhage and/or perforation in complex compared to simple appendicitis (**Figure S1**). Absolute counts of viable mononuclear (CD45^+^) cells were determined and an overall significant decrease of mononuclear cells was observed in all individual layers of the appendix of children with complex compared to simple appendicitis (**Figures 1A, B**). Further immune phenotyping showed that this affected T cells (CD3^+^) in all individual layers (epithelium simple appendicitis median 440x10^3^ cm^-2^, IQR 290-660x10^3^ cells cm^-2^ versus complex appendicitis 14x10^3^ cm^-2^, IQR 6.6-75x10^3^ cm^-2^; lamina propria 1200x10^3^ cm^-2^, IQR 520-1900x10^3^ cm^-2^ versus 64x10^3^ cm^-2^, IQR 13-180x10^3^ cm^-2^ cells cm^-2^; muscular layer 120x10^3^ cm^-2^, IQR 97-230x10^3^ cm^-2^ versus 9.5 x10^3^ cm^-2^, IQR 3.8-51 x10^3^ cm^-2^) (**Figures 1A, B**). Analyses of CD4^+^ and CD8^+^ T cells showed a trend towards reduced frequencies of CD4^+^ T cells in complex compared to simple appendicitis, but this did not reach significance (**Figures 1C, D**). In sum, the appendix of children with complex appendicitis showed cytopenia, with specifically reduced absolute numbers of T cells compared to simple appendicitis.

### Increased frequencies of differentiated CD4^+^ T cells and CD8^+^ T cells in the appendix of children with complex appendicitis

**Figure 2:**
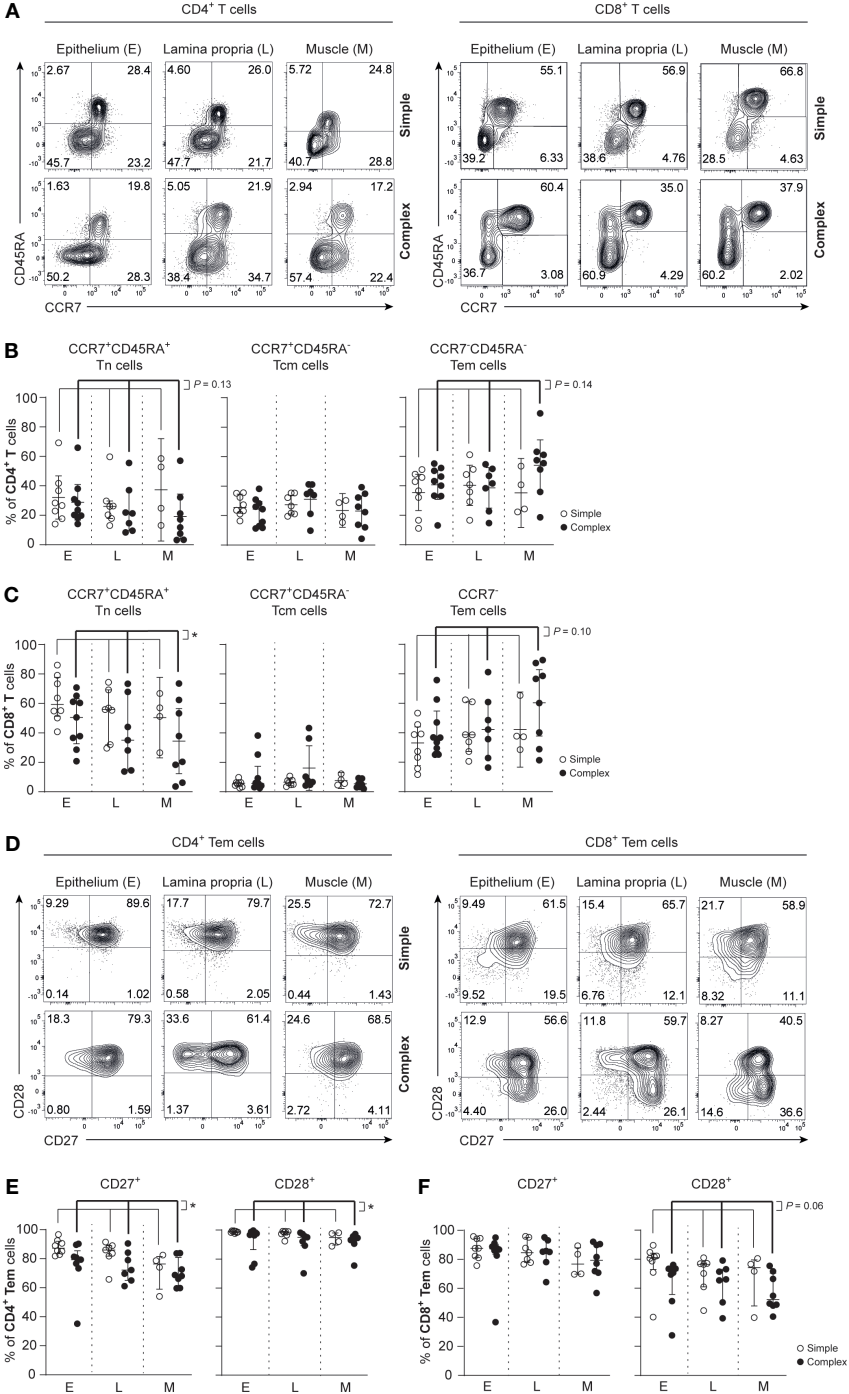
Disease severity is associated with an increased effector phenotype in CD4^+^ T cells and CD8^+^ T cells in appendicitis in children. **(A)** Gating strategy used to determine CCR7 and CD45RA expression on CD4^+^ T cells and CD8^+^ T cells. **(B)** Frequencies (%) of CD4^+^ Tn cells: naïve (CCR7^+^ CD45RA^+^); Tcm cells: central memory (CCR7^+^ CD45RA^-^); and Tem cells: effector memory (CCR7^-^ CD45RA^-^) in epithelium **(E)**, lamina propria (L) and muscular (M) layers. **(C)** Frequencies (%) of CD8^+^ Tn cells: naïve (CCR7^+^ CD45RA^+^); Tcm cells: central memory (CCR7^+^ CD45RA^-^); and Tem cells: effector memory (CCR7^-^ CD45RA^+/-^) in all three tissue layers. **(D)** CD27 and CD28 expression by CD4^+^ T cells and CD8^+^ Tem cells. **(E)** Frequencies (%) of CD27 and CD28-expression by CD4^+^ Tem cells in all three tissue layers. **(F)** Frequencies (%) of CD27 and CD28 expression by CD8^+^ Tem cells in all three tissue layers. **(B, C, E, F)** represent appendiceal epithelium (simple appendicitis n = 8 and complex appendicitis n = 9), lamina propria (simple appendicitis n = 7 and complex appendicitis n = 7) and muscular layer (simple appendicitis n = 4 and complex appendicitis n = 8). In **(F)** n = 8 for Tem cells in complex appendicitis in the epithelium due to lack of cells (<50) for subset gating. Representative flow cytometric plots are shown. Errors bars represent median percentage with ± interquartile range (IQR). All two-way ANOVA comparisons with Bonferroni correction. *P <.05.

Differentiated effector T cells are known potent producers of cytokines and contribute to inflammation (26, 27). In all tissue layers of the appendix combined, a trend towards decreased frequencies of naïve CD4^+^ T cells (Tn; CCR7^+^CD45RA^+^) was observed in children with complex compared to simple appendicitis (**Figures 2A, B**). In line, a trend towards increased frequencies of effecter memory CD4^+^ T cells (Tem; CCR7^-^CD45RA^-^) was observed in all layers of the appendix combined in children with complex compared to simple appendicitis. The CD8^+^ T cell compartment was similarly affected with a decrease of CD8^+^ Tn cells (CCR7^+^CD45RA^+^) and a trend towards increased CD8^+^ Tem cells (CCR7^-^CD45RA^+/-^) in all layers of the appendix combined in children with complex appendicitis (**Figures 2A, C**).

Assessment of CD27 and CD28 on T cell populations in the appendix revealed that CD4^+^ Tem cells had reduced CD27 expression in all layers of the appendix combined in children with complex compared to simple appendicitis (epithelium simple appendicitis median 87%, IQR 85-91% versus complex appendicitis median 79%, IQR 77-81%; lamina propria median 86%, IQR 83-88% versus median 72%, IQR 67-82%; muscular layer median 76%, IQR 69-80% versus median 68%, IQR 65-75%). And to a lesser extent, CD4^+^ Tem cells had a reduced expression of CD28 in all layers of the appendix combined in children with complex appendicitis (**Figures 2D, E**). In the CD8^+^ T cell compartment, a trend towards a reduced CD28 expression on CD8^+^ Tem cells was observed in all layers of the appendix combined of children with complex compared to simple appendicitis (epithelium simple appendicitis median 81%, IQR 77-83% versus complex appendicitis median 71%, IQR 51-74%; lamina propria median 77%, IQR 66-77% versus median 66%, IQR 58-72%; muscular layer median 74%, IQR 64-78% versus median 52%, IQR 49-68%) (**Figures 2D, F**). These findings indicate that T cells in the appendix, including CD4^+^ Tem cells specifically, exhibit an increased differentiation status in children with complex appendicitis.

### CD69^+^CD4^+^ Tem cells and CD69^+^CD8^+^ Tem cells are reduced in the appendix of children with complex appendicitis

**Figure 3:**
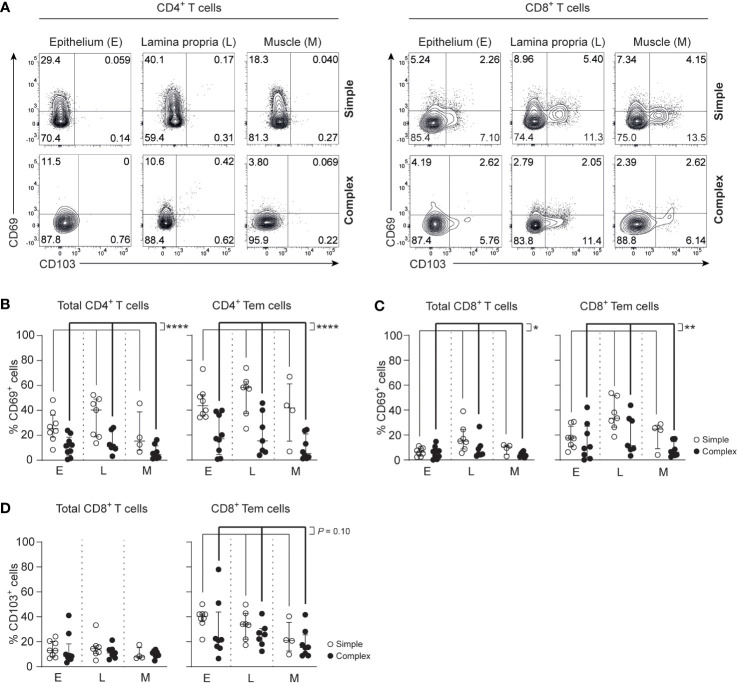
Severity of appendicitis is associated with a decrease of Trm CD4^+^ T cells and Trm CD8^+^ T cells. **(A)** CD69 and CD103 expression on CD4^+^ and CD8^+^ T cells. **(B)** Frequencies (%) of CD69 expression by CD4^+^ T cells and CD4^+^ effector memory T cells (Tem cells) in the epithelium (E), lamina propria (L) and muscular (M) layers. **(C)** Frequencies (%) of CD69^+^ CD8^+^ T cells and CD69^+^ CD8^+^ Tem cells in all three tissue layers. **(D)** Frequencies (%) of CD103^+^ CD8^+^ T cells and CD103^+^ CD8^+^ Tem cells in all three tissue layers. **(B–D)** represent appendiceal epithelium (simple appendicitis n = 8 and complex appendicitis n = 9), lamina propria (simple appendicitis n = 7 and complex appendicitis n = 7) and muscular layers (simple appendicitis n = 4 and complex appendicitis n = 8). In **(C, D)** n = 8 for epithelial Tem cells in complex appendicitis due to lack of cells (<50) for subset gating. Representative flow cytometric plots are shown. Errors bars represent median percentage with ± interquartile range (IQR). All two-way ANOVA comparisons with Bonferroni correction. *P<.05, **P<.01 and ****P<.0001.

Tissue-resident memory T cells (Trm) that express CD69 and/or CD103 are a hallmark of physiological immunity against pathogens and support the maintenance of a healthy mucosal barrier (18, 28). At the same time, dysregulation of Trms can contribute to inflammation (20, 21). CD4^+^ Trm cells (CCR7^-^CD45RA^-^CD69^+^) were decreased in all layers of the appendix combined in children with complex compared to simple appendicitis (epithelium simple appendicitis median 44%, IQR 37-51% versus complex appendicitis median 17%, IQR 8-36%; lamina propria median 58%, IQR 47-61% versus median 15%, IQR 7-33%; muscular layer median 42%, IQR 32-50% versus median 5%, IQR 2-16%) (**Figures 3A, B**). Similarly, CD8^+^ Trm cells (CCR7^-^CD45RA^+/-^CD69^+^) were decreased in all layers of the appendix combined in children with complex compared to simple appendicitis (epithelium simple appendicitis median 18%, IQR 12-21% versus complex appendicitis median 10%, IQR 3-27%; lamina propria median 34%, IQR 29-45% versus median 11%, IQR 9-32%; muscular layer median 25%, IQR 19-26% versus median 6%, IQR 4-11%) (**Figures 3A, C**). In addition, a trend towards a decreased expression of CD103 on CD8^+^ Tem cells was demonstrated in all layers of the appendix combined of children with complex compared to simple appendicitis (**Figures 3A, D**). Taken together, complex appendicitis is characterized by an overall loss of T cells and in particular a decrease in the frequencies of Trm cells in the appendix.

### Increased IL-17A production by appendiceal epithelial CD4^+^ T cells in children with complex appendicitis

**Figure 4:**
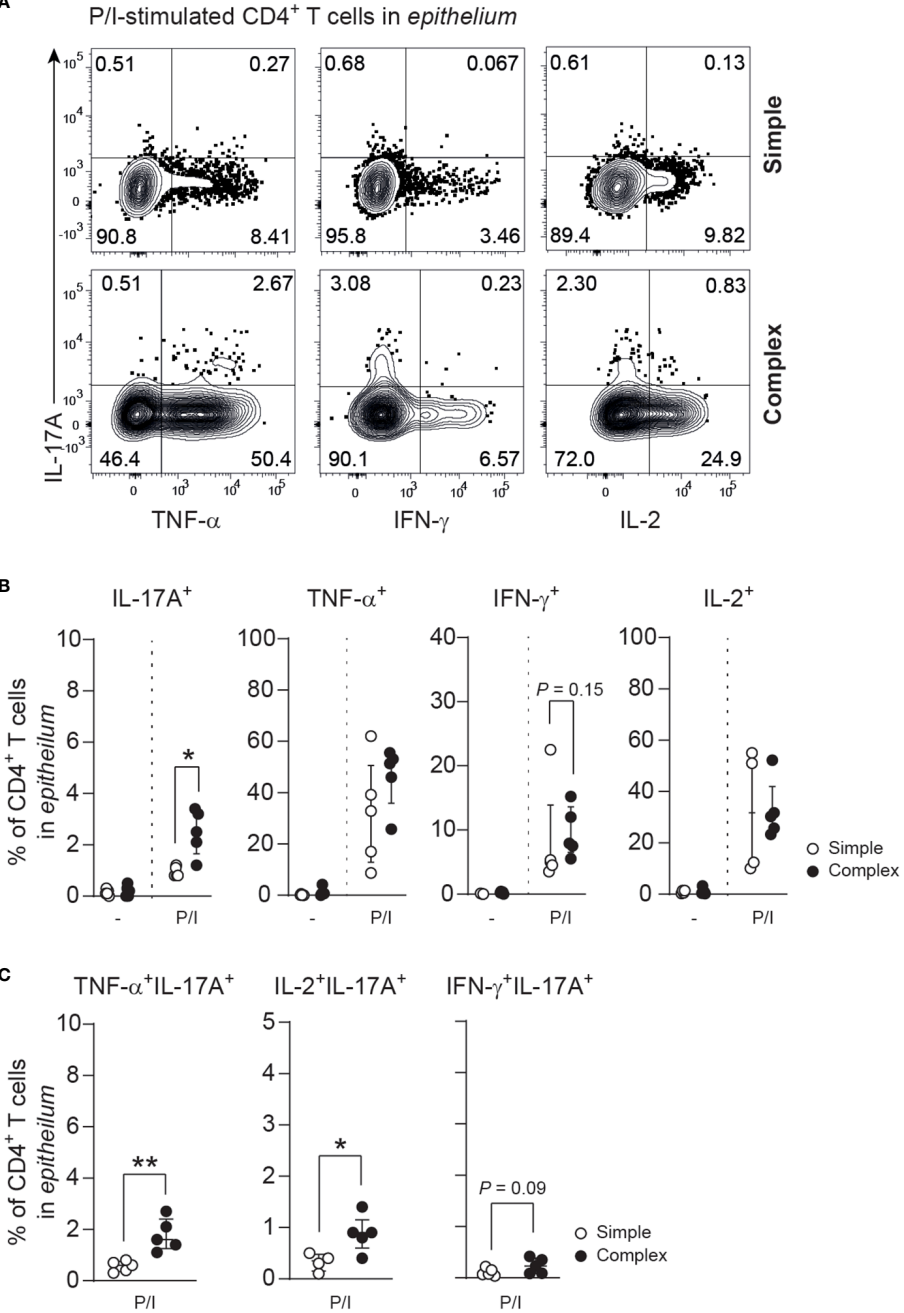
Severity of appendicitis is associated with increase of IL-17A-producing CD4^+^ T cells. **(A)** IL-17A, TNF, IFN-γ and IL-2 production in epithelial CD4^+^ T cells after P/I-stimulation. **(B)** Frequencies (%) of IL-17A, TNF, IFN-γ and IL-2 producing epithelial CD4^+^ T cells. **(C)** Frequencies (%) of cytokine co-production (IL-17A with TNF, IFN-γ or IL-2) by epithelial CD4^+^ T cells. **(B, C)** represent n = 5 for simple appendicitis and n = 5 for complex appendicitis in appendiceal epithelium, except for IL-2 where n = 4 for simple appendicitis. Representative flow cytometric plots are shown. Errors bars represent median percentage with ± interquartile range (IQR). All Mann-Whitney U comparisons. *P <.05 and **P <.01.

**Figure 5:**
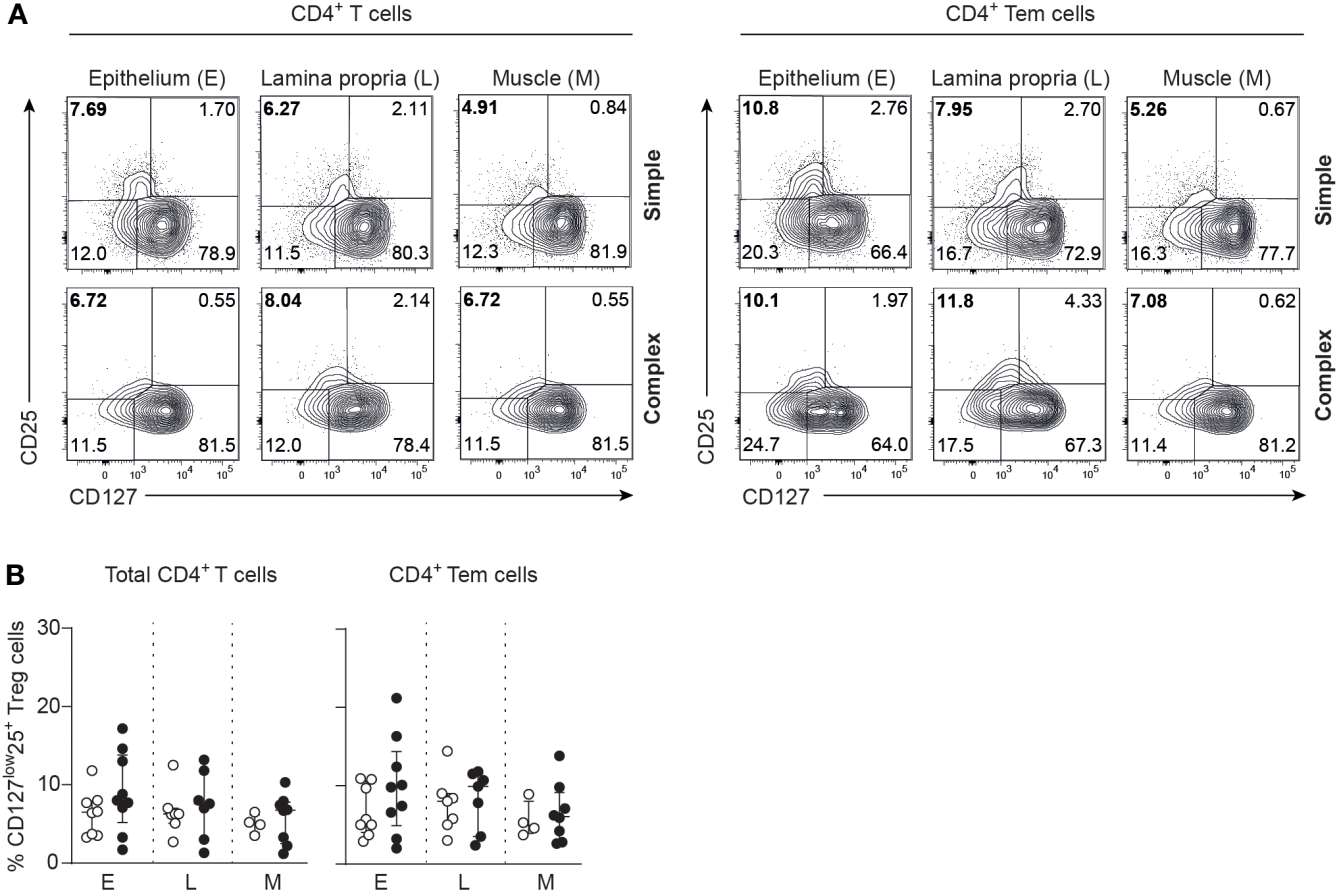
Comparable frequencies of CD4^+^ Tregs in simple and complex appendicitis. **(A)** CD25 and CD127 expression by CD4^+^ T cells and CD4^+^ Tem cells. **(B)** Frequencies (%) of CD127^low^CD25^+^ within CD4^+^ T cells and CD4^+^ Tem cells (Tregs) in the epithelium (E) (simple appendicitis n = 8 and complex appendicitis n = 9), lamina propria (L) (simple appendicitis n = 7 and complex appendicitis n = 7) and muscular (M) layers (simple appendicitis n = 4 and complex appendicitis n = 8). Representative flow cytometric plots are shown. Errors bars represent median percentage with ± interquartile range (IQR). Two-way ANOVA comparison with Bonferroni correction showed no significant difference in Tregs between simple and complex appendicitis.

Skewing of T helper responses and cytokine production is a feature of several other intestinal inflammatory diseases (14, 16, 22, 29, 30). IL-17A production of epithelial CD4^+^ T cells derived from the appendix of children with complex compared to simple appendicitis was increased upon P/I-stimulation (**Figures 4A, B**). Furthermore, a trend towards an increased production of interferon-gamma (IFN-γ) was observed from appendiceal epithelial CD4^+^ T cells of children with complex appendicitis. In addition, IL-17A co-production with TNF and IL-2 by appendiceal epithelial CD4^+^ T upon P/I stimulation was increased in children with complex appendicitis (**Figures 4A, C**). At the same time, similar frequencies of CD4^+^ Tregs (CCR7^-^CD45RA^-^CD127^low^CD25^+^) were found in all layers of the appendix combined of children with simple and complex appendicitis (epithelium simple appendicitis median 5%, IQR 5-10% versus complex appendicitis median 10%, IQR 7-12%; lamina propria median 8%, IQR 5-9% versus median 10%, IQR 6-11%; muscular layer median 5%, IQR 4-6% versus median 6%, IQR 4-8%) (**Figures 5A, B**). Taken together, these findings demonstrate an increase of epithelial Th17 cells in the appendix of children with complex compared to simple appendicitis, whereas regulatory responses remain relatively unchanged under these inflammatory conditions.

### Th17 responses in complex appendicitis are associated with specific microbiota

**Figure 6:**
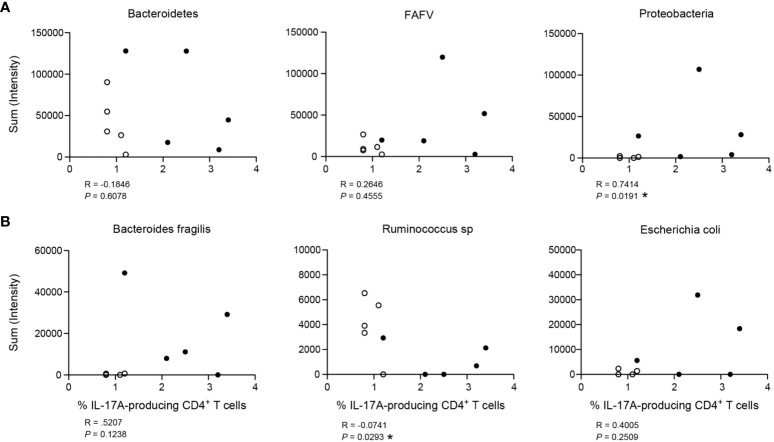
Association of IL-17A production by CD4^+^ T cells with bacterial load (intensity). **(A)** Three main phyla of bacteria: Bacteroidetes, FAFV (Firmicutes, Actinobacteria, Fusobacteria and Verrucobacteria) and Proteobacteria. **(B)** Representation of three species found relatively abundant in the appendix: *Bacteroides fragilis* (phylum of Bacteroidetes), *Ruminococcus sp* (phylum of Firmicutes) and *Escherichia coli* (phylum of Proteobacteria*)*. For both **(A)** and **(B)**, figures represent correlation of frequency (%) of IL-17A production by CD4^+^ T cells on x-axis with bacterial load (sum intensity in relative fluorescence units) on y-axis. Children with simple appendicitis (n = 5) are represented by white dots, children with complex appendicitis (n = 5) are represented by black dots. All Spearman correlations, *P<.05.

Microbes can drive T helper polarization, and in particular Th17 responses (31, 32). Our group recently showed that complex appendicitis is associated with compositional changes in microbiota compared to simple appendicitis (24). Using this unique data from the same cohort, we assessed whether IL-17A-production was associated with the three main phyla of bacteria. A positive correlation was detected between Proteobacteria and IL-17A production by appendiceal epithelial CD4^+^ T cells (R = 0.7414, P = 0.019), whereas there was no significant correlation with the phylum of Bacteroidetes or FAFV *(*Firmicutes, Actinobacteria, Fusobacteria and Verrucomicrobia) (R = -0.1846, P = 0.6078; and R = 0.2646, P = 0.4555 respectively) (**Figure 6A**). Next, we determined whether IL-17A production was associated with specific species that were abundant in the appendix, including *Escherichia coli, Bacteroides fragilis* and *Ruminococcus* sp. Of these, *Ruminococcus* sp. were negatively correlated with IL-17A production by epithelial CD4^+^ T cells (R = -0.0741, P = 0.0293). And, although higher quantities of both *Escherichia coli* and *Bacteroides fragilis* were detected in children with increased IL-17A production, this association did not reach significance (**Figure 6B**). Taking into account the limited number of samples, these data suggest that the microbial composition is associated with IL-17A production by epithelial CD4^+^ T cells in the appendix.

## Discussion

Here, we performed an in-depth analysis of appendiceal T cells in children with simple and complex appendicitis. Appendiceal CD4^+^ T cell populations in children with complex appendicitis were characterized by a decrease of molecules mediating tissue-residency and an increase of IL-17A production compared to children with simple appendicitis. Specifically, frequencies of dual cytokine producing Th17 cells were increased in the appendix of children with complex appendicitis. Furthermore, enrichment of Proteobacteria positively correlated to IL-17A^+^ CD4^+^ T cells, whereas *Ruminococcus* sp. negatively correlated with IL-17A^+^ CD4^+^ T cells in children with appendicitis. Taken together, these findings demonstrate a disruption of local T cells with a decrease of frequencies of tissue-resident memory T cells and increased Th17 responses in the appendix of children with complex compared to simple appendicitis, which were associated with intestinal microbial changes.

CD4^+^ Trm and CD8^+^ Trm cells (expressing CD69 and/or CD103) provide critical protection against pathogens (17, 18). But when dysregulated they can also contribute to intestinal inflammation (14, 16, 20–22). A lower percentage of appendiceal phenotypic Trms was detected in children with complex compared to simple appendicitis which may indicate a reduced protection against local microbes that can mediate inflammation in complex appendicitis. Although the decrease in absolute numbers of T cells in complex compared to simple appendicitis suggests a loss of Trms, based on these cross-sectional data, we cannot exclude that the change in percentage of CD69^+^ T cells is due to an influx of infiltrating T cells. Further studies are needed to assess the fate of Trms in simple and complex appendicitis.

Increased frequencies of IL-17A^+^ CD4^+^ T cells were observed in the epithelium of the appendix of children with complex appendicitis compared to simple appendicitis. IL-17 is a known key mediator of recruitment and activation of neutrophils during inflammation (33, 34) and in line we have previously reported increased numbers of neutrophils in complex appendicitis (13). Moreover, appendiceal Treg frequencies remained similar between complex and simple appendicitis. Thus, in complex appendicitis, production of IL-17A together with co-production with TNF and IL-2 by CD4^+^ T cells was increased, enhancing local inflammation. This augmented pro-inflammatory activity of CD4^+^ T cells in complex appendicitis is in line with the reduced expression of CD27 and CD28 indicating an increased effector phenotype of CD4^+^ T cells in the appendix of children with complex compared to simple appendicitis. CD4^+^ Tem cells with reduced expression of CD27 and CD28 have cytolytic capacities mimicking cytotoxic CD8^+^ T cells (35, 36). Moreover, CD27 and CD28 downregulation on CD4^+^ T cells has been previously associated with other intestinal inflammatory diseases such as Crohn’s Disease (37).

Although there is increasing evidence that simple appendicitis can be treated with antibiotics and may not require surgical intervention, identification of children with simple or complex appendicitis remains challenging (3–5). A biomarker derived from blood would provide a helpful instrument to aid the classification of appendicitis and identify children at risk for complex appendicitis requiring surgical intervention. To this end, previous studies analyzed immune parameters in peripheral blood samples of children with appendicitis: Peeters et al. detected in particular increased plasma levels of IL-6 in complicated (complex) compared to uncomplicated (simple) appendicitis. In support of these data Rubér et al. also detected increased levels of IL-6, IL-17, CCL2, MMP-8 and MMP-9 in gangrenous (complex) compared to phlegmonous (simple) appendicitis (38, 39). IL-6 and IL-1β are the main cytokines instructing Th17 cell polarization (40–42). These findings based on analyses of blood suggested that Th17 cells may play an important role in complex appendicitis. However, analyses of appendiceal tissues were lacking in these studies. The findings presented here address this knowledge gap and demonstrate an increased appendiceal (pathogenic) Th17 response in children with complex appendicitis, which corresponds to these previously observed blood-derived cytokine profiles. Peripheral blood samples were not collected within the same patient in this study to minimize the invasive procedures for children. Therefore, a correlation between plasma levels of IL-6, IL-1β, IL-17 and Th17 cells in the appendix was unfortunately not possible. In sum, the findings presented here together with data from previous studies suggest that aberrant Th17 responses are increased in complex appendicitis and plasma levels of IL-17A and IL-6 may offer potential biomarkers that should be investigated in future studies.

Dysregulation of the interplay between the microbiota and local tissue-resident immune responses underlies a variety of inflammatory diseases (11, 43, 44). Integration of previously published data characterizing the appendiceal microbiota in patients with appendicitis (24) with the above described immunophenotypic data, allowed to perform unique intra-patient analyses of intestinal microbiota and intestinal T cell analyses from the same location. The phylum of Proteobacteria positively correlated with IL-17A production by CD4^+^ T cells, whereas a negative correlation was observed with *Ruminococcus* spp. An increase of the phylum of Proteobacteria has been previously linked to disease severity in patients with Crohn’s disease (45). And although a direct correlation of IL-17A^+^ CD4^+^ T cells with *Escherichia coli* was not detected, *Escherichia coli*, a member of the phylum of Proteobacteria, and several of its strains have been shown to promote Th17-responses and inflammation (46). Although some studies have suggested a role of *Ruminococcus* sp. in tolerance (47), functional studies are needed to decipher microbe specific effects. Controlled *in vivo* experiments can help to next identify the role of specific microbiota in shaping local appendiceal T cell functionality and tissue inflammation in appendicitis models.

Further limiting the interpretation of the results is the relatively small sample size, and conclusions, specifically on IL-17A correlations with bacteria, should be interpreted with caution. Especially, as analyses of the effects of potential confounding factors, such as antibiotic regimes or age, could not be performed due to the small sample size. Of note, our group previously published analyses comparing appendiceal and rectal swab-derived microbiota in children with simple and complex appendicitis in a larger cohort, however without Th17 cells analyses (24, 48). In children with complex appendicitis the appendiceal microbiota was increased in terms of both diversity and intensity compared to simple appendicitis; and a greater similarity was observed between the appendiceal microbiota and microbiota analyzed with rectal swabs within children with complex compared to simple appendicitis; However, the power to distinguish between simple and complex appendicitis based on microbiota analyses from rectal swabs was unfortunately low. Importantly, specific antibiotic regimes did not seem to impact the microbiota. Presumably as the antibiotics were administered shortly before incision (24, 48). Nevertheless, we cannot exclude that the used antibiotics may have affected the local microbiota. This consideration should be kept in mind when interpreting results of the current study and it emphasizes the need for precise microbiota studies in animal appendicitis models.

In conclusion, findings of this study demonstrate disruption of local T cell responses in the appendix of children with complex compared to simple appendicitis, characterized by a decrease in frequencies of Trms and increased pathogenic Th17 responses associated with specific microbiota alterations. They provide a deeper understanding of immune dysregulation in complex appendicitis and identify Th17 responses as a potential critical modulator, which needs to be further investigated to develop diagnostic tools for the identification of children at risk for complex appendicitis and select optimal treatment strategies.

## Data availability statement

The datasets presented in this article are not readily available because Data can be made available upon request when in line with the original informed consent. Requests to access the datasets should be directed to madeleine.bunders@leibniz-liv.de.

## Ethics statement

The studies involving humans were approved by the medical ethics committee of the Amsterdam UMC, with local confirmation by the medical board of the Red Cross Hospital, Beverwijk. The studies were conducted in accordance with the local legislation and institutional requirements. Written informed consent for participation in this study was provided by the participants’ legal guardians/next of kin.

## Author contributions

S-MT: Conceptualization, Data curation, Formal Analysis, Investigation, Methodology, Project administration, Resources, Visualization, Writing – original draft. RS: Data curation, Formal Analysis, Investigation, Methodology, Visualization, Writing – review & editing. AD: Formal Analysis, Investigation, Writing – review & editing. RB: Conceptualization, Funding acquisition, Investigation, Project administration, Resources, Writing – review & editing. TD: Conceptualization, Formal Analysis, Investigation, Methodology, Writing – review & editing. AB: Conceptualization, Formal Analysis, Investigation, Methodology, Writing – review & editing. LP: Data curation, Formal Analysis, Investigation, Writing – review & editing. HC: Investigation, Resources, Writing – review & editing. HH: Supervision, Writing – review & editing, Conceptualization, Investigation. LV: Investigation, Resources, Supervision, Writing – review & editing. RG: Conceptualization, Funding acquisition, Investigation, Formal analysis, Methodology, Project administration, Resources, Supervision, Writing – review & editing, Formal Analysis. MB: Conceptualization, Formal Analysis, Funding acquisition, Investigation, Methodology, Resources, Supervision, Writing – review & editing.

## Supplementary material

Supplementary Figure 1 Macroscopic intraluminal appearance of the twenty tissue samples included for final analysis in this study. Tissues were separated into simple (top panel) and complex (bottom panel) appendicitis based on predefined parameters (perioperative findings and histopathological report).

## References

[1] Rothrock SG Pagane J. Acute appendicitis in children: emergency department diagnosis and management. Ann Emerg Med (2000) 36:39–51. doi: 10.1067/mem.2000.105658 10874234

[2] Carr NJ. The pathology of acute appendicitis. Ann Diagn Pathol (2000) 4(1):46–58. doi: 10.1016/S1092-9134(00)90011-X 10684382

[3] Bhangu A Søreide K Di Saverio S Assarsson JH Drake FT. Acute appendicitis: Modern understanding of pathogenesis, diagnosis, and management. Lancet (2015) 386(10000):1278–87. doi: 10.1016/S0140-6736(15)00275-5 26460662

[4] Gorter RR The S-MML Gorter-Stam MAW Eker HH Bakx R van der Lee JH. Systematic review of nonoperative versus operative treatment of uncomplicated appendicitis. J Pediatr Surg (2017) 52:1219–27. doi: 10.1016/j.jpedsurg.2017.04.005 28449821

[5] Svensson JF Patkova B Almström M Naji H Hall NJ Eaton S. Nonoperative treatment with antibiotics versus surgery for acute nonperforated appendicitis in children. Ann Surg (2015) 261(1):67–71. doi: 10.1097/SLA.0000000000000835 25072441

[6] Helling TS Soltys DF Seals S. Operative versus non-operative management in the care of patients with complicated appendicitis. Am J Surg (2017) 214(6):1195–200. doi: 10.1016/j.amjsurg.2017.07.039 28941724

[7] Randal Bollinger R Barbas AS Bush EL Lin SS Parker W. Biofilms in the large bowel suggest an apparent function of the human vermiform appendix. J Theor Biol (2007) 249:826–31. doi: 10.1016/j.jtbi.2007.08.032 17936308

[8] Kooij IA Sahami S Meijer SL Buskens CJ te Velde AA. The immunology of the vermiform appendix: a review of the literature. Clin Exp Immunol (2016) 186(1):1–9. doi: 10.1111/cei.12821 27271818 PMC5011360

[9] Randal Bollinger R Lou EM Palestrant D Love SD Lin SS Parker W. Human secretory immunoglobulin A may contribute to biofilm formation in the gut. Immunology (2003) 109(4):580–7. doi: 10.1046/j.1365-2567.2003.01700.x PMC1782994 12871226

[10] Hanson NB Lanning DK. Microbial induction of B and T cell areas in rabbit appendix. Dev Comp Immunol (2008) 32(8):980–91. doi: 10.1016/j.dci.2008.01.013 PMC2408667 18329710

[11] Donaldson GP Lee SM Mazmanian SK. Gut biogeography of the bacterial microbiota. Nat Rev Microbiol (2016) 14:20–32. doi: 10.1038/nrmicro3552 26499895 PMC4837114

[12] Watson Ng WS Hampartzoumian T Lloyd AR Grimm MC. A murine model of appendicitis and the impact of inflammation on appendiceal lymphocyte constituents. Clin Exp Immunol (2007) 150(1):169–78. doi: 10.1111/j.1365-2249.2007.03463.x PMC2219294 17680826

[13] Gorter RR Wassenaar ECE de Boer OJ Bakx R Roelofs JJTH Bunders MJ. Composition of the cellular infiltrate in patients with simple and complex appendicitis. J Surg Res (2017) 214:190–6. doi: 10.1016/j.jss.2017.02.062 28624043

[14] Powrie F. Gut reactions: Immune pathways in the intestine in health and disease. EMBO Mol Med (2012) 4(2):71–4. doi: 10.1002/emmm.201100197 PMC3376842 22278905

[15] Asseman C Fowler S Powrie F. Control of experimental inflammatory bowel disease by regulatory T cells. Am J Respir Crit Care Med (2000) 162(4 Pt 2):S185–9. doi: 10.1164/ajrccm.162.supplement_3.15tac9 11029392

[16] Zenewicz LA Antov A Flavell RA. CD4 T-cell differentiation and inflammatory bowel disease. Trends Mol Med (2009) 15(5):199–207. doi: 10.1016/j.molmed.2009.03.002 19362058

[17] Masopust D Soerens AG. Tissue-resident T cells and other resident leukocytes. Annu Rev Immunol (2019) 37:521–46. doi: 10.1146/annurev-immunol-042617-053214 PMC7175802 30726153

[18] Cibrián D Sánchez-madrid F. CD69 : from activation marker to metabolic gatekeeper. Eur J Immunol (2019) 47(6):946–53. doi: 10.1002/eji.201646837 PMC6485631 28475283

[19] Schreiner D King CG. CD4+ memory T cells at home in the tissue: Mechanisms for health and disease. Front Immunol (2018) 9:2394. doi: 10.3389/fimmu.2018.02394 30386342 PMC6198086

[20] Bishu S El Zaatari M Hayashi A Hou G Bowers N Kinnucan J. CD4+ Tissue-resident memory T cells expand and are a major source of mucosal tumour necrosis factor α in active crohn’s disease. J Crohn’s Colitis (2019) 13(7):905–15. doi: 10.1093/ecco-jcc/jjz010 PMC6939878 30715262

[21] Hou G Bishu S. Th17 cells in inflammatory bowel disease: an update for the clinician. Inflammation Bowel Dis (2020) 26(5):653–61. doi: 10.1093/ibd/izz316 PMC11491631 31970388

[22] Schreurs RRCE Baumdick ME Sagebiel AF Kaufmann M Mokry M Klarenbeek PL. Human fetal TNF-α-cytokine-producing CD4 + Effector memory T cells promote intestinal development and mediate inflammation early in life. Immunity (2019) 50(2):462–476.e8. doi: 10.1016/j.immuni.2018.12.010 30770246

[23] Schreurs RRCE Drewniak A Bakx R Corpeleijn WE Geijtenbeek THB van Goudoever JB. Quantitative comparison of human intestinal mononuclear leukocyte isolation techniques for flow cytometric analyses. J Immunol Methods (2017) 445:45–52. doi: 10.1016/j.jim.2017.03.006 28274838

[24] The SML Bakx R Budding AE de Meij TGJ van der Lee JH Bunders MJ. Microbiota of children with complex appendicitis: different composition and diversity of the microbiota in children with complex compared with simple appendicitis. Pediatr Infect Dis J (2019) 38(10):1054–60. doi: 10.1097/INF.0000000000002434 31568143

[25] Budding AE Grasman ME Lin F Bogaards JA Soeltan-Kaersenhout DJ Vandenbroucke-Grauls CMJE. IS-pro: high-throughput molecular fingerprinting of the intestinal microbiota. FASEB J (2010) 24:4556–64. doi: 10.1096/fj.10-156190 20643909

[26] Moro-García MA Mayo JC Sainz RM Alonso-Arias R. Influence of inflammation in the process of T lymphocyte differentiation: Proliferative, metabolic, and oxidative changes. Front Immunol (2018) 9:339. doi: 10.3389/fimmu.2018.00339 29545794 PMC5839096

[27] Sallusto F Lenig D Förster R Lipp M Lanzavecchia A. Two subsets of memory T lymphocytes with distinct homing potentials and effector functions. Nature (1999) 401(6754):708–12. doi: 10.1038/44385 10537110

[28] Walsh DA Borges da Silva H Beura LK Peng C Hamilton SE Masopust D. The functional requirement for CD69 in establishment of resident memory CD8 + T cells varies with tissue location. J Immunol (2019) 203(4):946–55. doi: 10.4049/jimmunol.1900052 PMC6684481 31243092

[29] Van Der Vliet HJJ Nieuwenhuis EE. IPEX as a result of mutations in FOXP3. Clin Dev Immunol (2007) 2007:89017. doi: 10.1155/2007/89017 18317533 PMC2248278

[30] Charbonnier LM Janssen E Chou J Ohsumi TK Keles S Hsu JT. Regulatory T-cell deficiency and immune dysregulation, polyendocrinopathy, enteropathy, X-linked-like disorder caused by loss-of-function mutations in LRBA. J Allergy Clin Immunol (2015) 135(1):217–27. doi: 10.1016/j.jaci.2014.10.019 PMC4289093 25468195

[31] Lee N Kim WU. Microbiota in T-cell homeostasis and inflammatory diseases. Exp Mol Med (2017) 49(5):e340. doi: 10.1038/emm.2017.36 28546563 PMC5454441

[32] Di Gangi A Di Cicco ME Comberiati P Peroni DG. Go with your gut: the shaping of T-cell response by gut microbiota in allergic asthma. Front Immunol (2020) 11:1485. doi: 10.3389/fimmu.2020.01485 32760404 PMC7372123

[33] Griffin GK Newton G Tarrio ML Bu D Maganto-Garcia E Azcutia V. IL-17 and TNF-α sustain neutrophil recruitment during inflammation through synergistic effects on endothelial activation. J Immunol (2012) 188:6287–99. doi: 10.4049/jimmunol.1200385 PMC3370121 22566565

[34] Ge Y Huang M Yao Y-M. Biology of interleukin-17 and its pathophysiological significance in sepsis. Front Immunol (2020) 11:1558. doi: 10.3389/fimmu.2020.01558 32849528 PMC7399097

[35] Appay V Zaunders JJ Papagno L Sutton J Jaramillo A Waters A. Characterization of CD4 + CTLs ex vivo. J Immunol (2002) 168(11):5954–8. doi: 10.4049/jimmunol.168.11.5954 12023402

[36] Appay V Van Lier RAW Sallusto F Roederer M. Phenotype and function of human T lymphocyte subsets: Consensus and issues. Cytom A (2008) 73(11):975–83. doi: 10.1002/cyto.a.20643 18785267

[37] García De Tena J Manzano L Leal JC San Antonio E Sualdea V Álvarez-Mon M. Active Crohn’s disease patients show a distinctive expansion of circulating memory CD4+CD45RO+CD28null T cells. J Clin Immunol (2004) 24(2):185–96. doi: 10.1023/B:JOCI.0000019784.20191.7f 15024186

[38] Rubér M Andersson M Petersson BF Olaison G Andersson RE Ekerfelt C. Systemic Th17-like cytokine pattern in gangrenous appendicitis but not in phlegmonous appendicitis. Surgery (2010) 147(3):366–72. doi: 10.1016/j.surg.2009.09.039 19892382

[39] Peeters T Martens S D’Onofrio V Stappers MHT van der Hilst JCH Houben B. An observational study of innate immune responses in patients with acute appendicitis. Sci Rep (2020) 10(1):17352. doi: 10.1038/s41598-020-73798-3 33060696 PMC7562899

[40] Kimura A Kishimoto T. IL-6: regulator of treg/th17 balance. Eur J Immunol (2010) 40(7):1830–5. doi: 10.1002/eji.201040391 20583029

[41] Maynard CL Weaver CT. Intestinal effector T cells in health and disease. Immunity (2009) 31(3):389–400. doi: 10.1016/j.immuni.2009.08.012 19766082 PMC3109492

[42] Acosta-Rodriguez EV Napolitani G Lanzavecchia A Sallusto F. Interleukins 1beta and 6 but not transforming growth factor-beta are essential for the differentiation of interleukin 17-producing human T helper cells. Nat Immunol (2007) 8(9):942–9. doi: 10.1038/ni1496 17676045

[43] Daniels L Budding AE de Korte N Eck A Bogaards JA Stockmann HB. Fecal microbiome analysis as a diagnostic test for diverticulitis. Eur J Clin Microbiol Infect Dis (2014) 33:1927–36. doi: 10.1007/s10096-014-2162-3 24894339

[44] Dieterich W Schink M Zopf Y. Microbiota in the gastrointestinal tract. Med Sci (2018) 6:116. doi: 10.3390/medsci6040116 PMC6313343 30558253

[45] Vester-Andersen MK Mirsepasi-Lauridsen HC Prosberg MV Mortensen CO Träger C Skovsen K. Increased abundance of proteobacteria in aggressive Crohn’s disease seven years after diagnosis. Sci Rep (2019) 9(1):13473. doi: 10.1038/s41598-019-49833-3 31530835 PMC6748953

[46] Viladomiu M Kivolowitz C Abdulhamid A Dogan B Victorio D Castellanos JG. IgA-coated E. Coli enriched in Crohn’s disease spondyloarthritis promote TH17-dependent inflammation. Sci Transl Med (2017) 9(376):eaaf9655. doi: 10.1126/scitranslmed.aaf9655 28179509 PMC6159892

[47] Shimokawa C Kato T Takeuchi T Ohshima N Furuki T Ohtsu Y. CD8+ regulatory T cells are critical in prevention of autoimmune-mediated diabetes. Nat Commun (2020) 11(1):1922. doi: 10.1038/s41467-020-15857-x 32321922 PMC7176710

[48] The S-MML de Meij TGJ Budding AE Bakx R van der Lee JH Poort L. The potential of rectal swabs to differentiate simple and complex appendicitis in children with a microbiota-based test. Eur J Pediatr (2022) 181(12):4221–6. doi: 10.1007/s00431-022-04627-0 PMC9649451 36195698

