# Supplementary figures and images for: Enhanced Th17 responses in the appendix of children with complex compared to simple appendicitis are associated with microbial dysbiosis
Source: Front Immunol. 2024 Jan 4;14:1258363. doi: 10.3389/fimmu.2023.1258363 (PMC10794624; doi:10.3389/fimmu.2023.1258363)

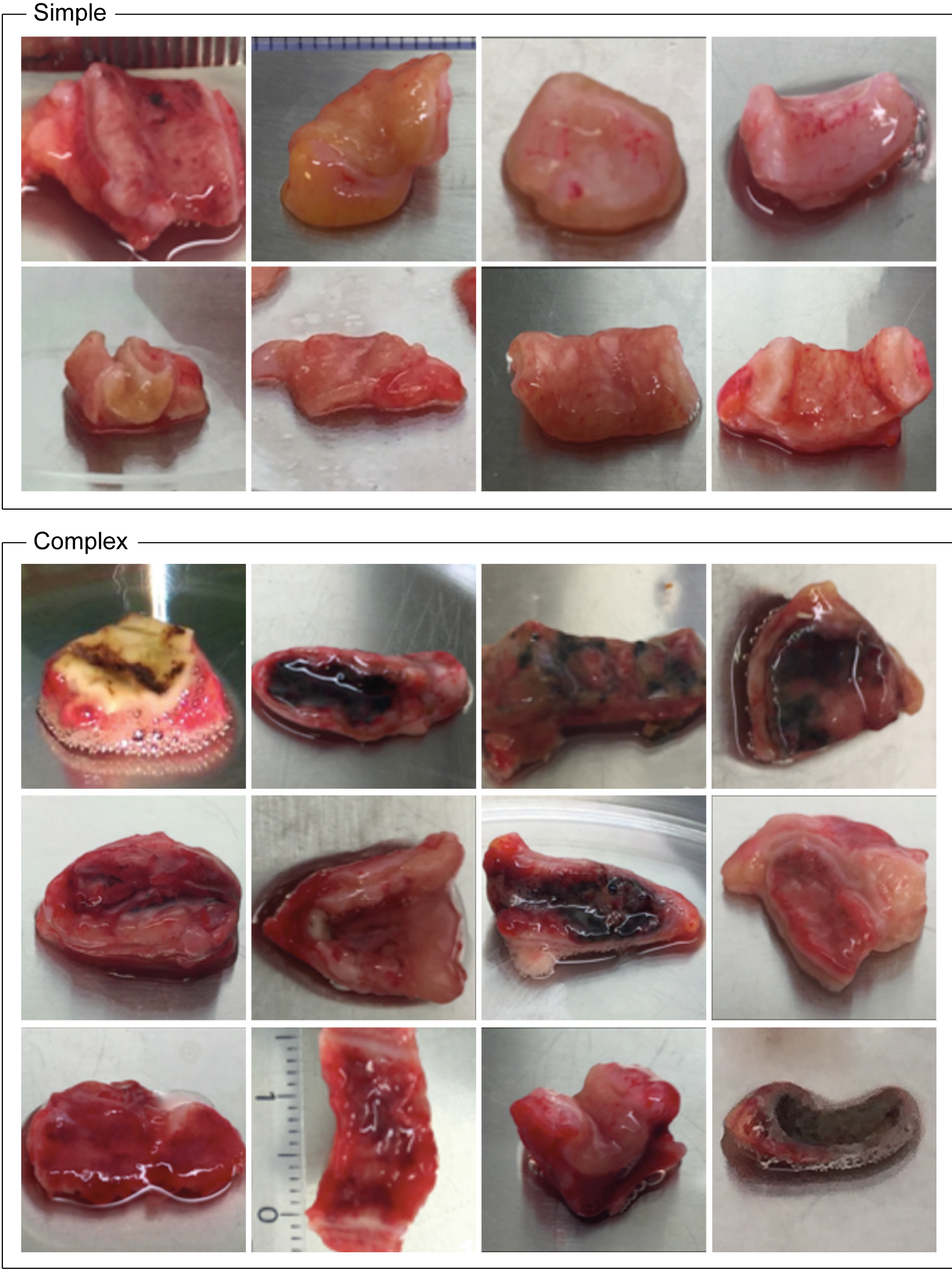

Supplement: Supplementary Figure 1 — Macroscopic intraluminal appearance of the twenty tissue samples included for final analysis in this study. Tissues were separated into simple (top panel) and complex (bottom panel) appendicitis based on predefined parameters (perioperative findings and histopathological report). [file Image_1.tiff]
